# Unpacking the notion of “serious” genetic conditions: towards implementation in reproductive decision-making?

**DOI:** 10.1038/s41431-024-01681-0

**Published:** 2024-08-10

**Authors:** Erika Kleiderman, Felicity Boardman, Ainsley J. Newson, Anne-Marie Laberge, Bartha Maria Knoppers, Vardit Ravitsky

**Affiliations:** 1 Department of Social and Preventive Medicine, School of Public Health, University of Montreal, Montreal, QC Canada; 2 Division of Health Sciences, Warwick Medical School, University of Warwick, Coventry, UK; 3 Faculty of Medicine and Health, Sydney School of Public Health, Sydney Health Ethics, The University of Sydney, Sydney, NSW Australia; 4 Medical Genetics Division, Department of Pediatrics, Centre Hospitalier Universitaire Sainte-Justine and Université de Montréal, Montreal, QC Canada; 5 Centre of Genomics and Policy, Department of Human Genetics, Faculty of Medicine, McGill University, Montreal, QC Canada; 6 The Hastings Center, Garrison, NY USA

**Keywords:** Ethics, Genetics, Medical ethics, Public health

## Abstract

The notion of a “serious” genetic condition is commonly used in clinical contexts, laws, and policies to define and delineate both the permissibility of and, access to, reproductive genomic technologies. Yet, the notion lacks conceptual and operational clarity, which can lead to its inconsistent appraisal and application. A common understanding of the relevant considerations of “serious” is lacking. This article addresses this conceptual gap. We begin by outlining existing distinctions around the notion of “serious” that will factor into its appraisal and need to be navigated, in the context of prenatal testing and the use of reproductive genomic technologies. These include tensions between clinical care and population health; the impact of categorizing a condition as “serious”; and the role of perception of quality of life. We then propose a set of four core dimensions and four procedural elements that can serve as a conceptual tool to prompt a mapping of the features of seriousness in any given context. Ultimately, consideration of these core dimensions and procedural elements may lead to improvements in the quality and consistency of decision-making where the seriousness of a genetic condition is a pivotal component at both a policy and practice level.

## Introduction

The seriousness of a condition is a complex and multi-layered concept that is frequently used to define and delineate the scope of ethically appropriate and/or legally permissible clinical applications of reproductive genomic technologies (e.g., pre-implantation genetic testing—PGT, non-invasive prenatal testing—NIPT, prenatal diagnosis, and population screening, among others). In many jurisdictions, “serious” serves as an important criterion for resource distribution and priority setting in healthcare systems (e.g., France, the Netherlands, Norway, Sweden, and the United Kingdom) [1, 2]. While not the subject of this article, it bears noting that policy discussions around human germline genome editing also emphasize the notion of “serious” genetic conditions as an important criterion or threshold in determining: (a) the permissibility for a translational path from bench to bedside, as well as (b) potential candidate genetic conditions for such technologies [3–9].

Yet even today, “serious” remains vague and ill-defined. This lack of conceptual and operational clarity renders its application challenging and decision-making potentially arbitrary, and highly variable across social and cultural contexts. Several authors have noted this wide variation in application and alluded to the challenges that emerge when a vague and biomedically determined conceptualization of “serious” is practically applied as a threshold/filter of permissibility or access to genomic technologies [10–15].

Alongside differing interpretations of meaning, the words “serious” and “severe” are often employed interchangeably. Although both are used to describe the intensity, extent, or degree of something, they are often used in somewhat different contexts and with different nuances. The Merriam-Webster dictionary defines “serious” as “having important or dangerous possible consequences” (e.g., a serious illness) and “severe” as “very painful or harmful” or “of a great degree” (e.g., severe pain) [16, 17]. “Serious”; therefore, appears to encompass the qualitative and experiential aspects of a genetic condition, while “severe” appears to imply a quantitative measure of intensity along a gradient (i.e., the measurable traits and criteria that comprise the biomedical aspects or clinical manifestations of a condition). In our view, these concepts cannot be completely separated, and each brings value to decision-making depending on context. A full consideration of the relationship between these concepts is beyond the scope of this article and we have opted to use “serious” for the reasons outlined above.

Attempts have been made to operationalize seriousness through various measures of the condition(s) in question (e.g., the intensity, frequency, and duration of symptoms; disease-specific scales; functional impairment; quality of life indicators; prognostic indicators) [18–21]. These are typically technical measures, with a focus on what are considered to be the objective elements of a genetic condition, with little consideration of the perspectives of those living with the condition, which may differ substantially from biomedical perspectives [10, 22]. Indeed, these accounts are always filtered through an individual’s social, cultural, economic, and environmental context, with each having a significant impact on their lived experiences [22].

However, the assessment of seriousness remains inherently value-laden and contested, and in many policy contexts is purposefully left open-ended. If the current utilization of seriousness as a filter for access to prenatal testing and reproductive genomic technologies is to be justified, there needs to be a common understanding of the factors that must be considered in order to arrive at a shared understanding of the various features of this concept, and to ensure a consistent and coherent appraisal of seriousness that is responsive to different contexts.

Based on the literature and our previous work [23, 24], the appraisal of seriousness must address and navigate existing distinctions and tensions. In the context of prenatal testing and the use of reproductive genomic technologies, these include the tension between clinical care and population health; the impacts of categorizing a condition as “serious”; and the perception of quality of life.

### Tension between clinical care and population health

In clinical care, patients (i.e., prospective parents) together with their healthcare providers, make decisions about reproductive and prenatal testing options based on the would-be parent’s understanding of the foetal diagnosis, their needs, their values, and their familial/personal context [23, 25, 26]. The focus here is on subjective and contextual elements that impact patient well-being. Therefore, relying solely on an objective or biomedical qualification of “serious” in clinical care would be dismissive of the person’s experience and fail to account for a variety of factors that may influence their understanding of seriousness, as well as concepts such as health and disease (e.g., ethnocultural and spiritual-religious) [23]. In population health, assessments of a genetic condition’s seriousness are typically made in aggregate and ex ante, based on a medical model (i.e., seriousness as the objective, quantifiable, and measurable qualifier). Decisions are applied in a top–down manner, so as to foster a more consistent and coherent application of laws, regulations, and policies. Such decisions require operational definitions and more consensual perspectives [23]. Population health policy and its implementation are undertaken with specific resources, targets, and groups of individuals (i.e., sub-populations/communities) in mind. The western paradigm has been predominant in this context, with a focus on objective, measurable outcomes, which often involves the use of more collectively focused criteria and measures to ensure an equitable and justified use of resources. Governments may also have to address conflicting interests between different lobbying patient organizations, even when they represent the same condition. In this context, the focus is on the prevalence of a genetic condition in a given population and on the potential impacts for the population (e.g., funding for and equitable access to screening, treatments, or to social support), as well as improving the overall health of the population, while also promoting plural values and upholding reproductive autonomy (e.g., PGT; NIPT; carrier screening; newborn screening) [24, 27]. The number and type of genetic conditions deemed to be “serious” enough to offer a population-scale intervention will likely be narrower than conditions for which testing may be offered in clinical care (e.g., prenatal screening for common aneuploidies versus prenatal testing for a rare genetic condition). It is the ability of clinical care to attend to and incorporate the context of an individual and their circumstances into decision-making that allows for the broader scope and number of conditions deemed “serious”.

### Impacts of categorizing a condition as “serious”

Seriousness is an inherently subjective term and is shaped by each person’s unique circumstances (i.e., values, coping mechanisms, perceptions of the world around them, and physical environment). Therefore, it is essential to be mindful of what the aim is when it comes to labelling a condition as “serious” in a given context. In most cases, the aim is to play a gatekeeping role in the management of resources and to determine who gets access to novel reproductive genomic technologies. Kukla argues that “it is appropriate to categorize a condition as a disease when it serves legitimate strategic goals to at least partially medicalize that condition, and when the condition is pathological from inside the epistemology and metaphysics of medicine” (p. 131) [28]. Such an approach allows for the evolution of the concept and consideration of contextual aspects.

We recognize that prenatal genomic testing programs (including PGT and NIPT) for specific medically defined genetic conditions can be contentious. For some, it may feel, based on their experience, that their condition is not “serious” (or considered a pathology at all) and hence does not warrant prenatal testing or interventions (e.g., deafness or achondroplasia) [29, 30]. Several features of the experience with a genetic condition have been linked to this view, some linked to the impairment itself (including age of onset or whether it is degenerative), as well as personal, social, cultural, and environmental factors such as access to resources, structural development, and stigma [10, 22]. Indeed, beyond developing a common understanding of seriousness, it is essential to grasp that the categorization of a condition as “serious” can have wide-ranging consequences beyond those initially desired. For example, there is concern that seriousness may be instrumentalized to impose moral duties and obligations on populations to (possibly coercively) follow a preferred course of action (e.g., to not have certain kinds of children or to undergo a specific prenatal test) [31]. The inclusion of a condition on a list accepted for offer of PGT or antenatal screening can have an implicit message to the population that this condition must be “serious” if the government offers access to tests for it, particularly within resource constrained healthcare systems [32, 33]. Such an approach often overemphasizes the loss of opportunity and disadvantages experienced by those living with genetic conditions (e.g., the need to reduce suffering/burden of the condition), while ignoring the existing social and environmental barriers that prevent full participation in daily life, and further reinforce inequalities in access to appropriate healthcare and resources [34].

In addition to having consequences beyond those initially desired, labelling a condition can have both beneficial and harmful implications. Beneficial in that it may provide feelings of validation and allow greater access to treatment and support (emotional, social, and financial), or potentially harmful in that it may lead to negative psychological consequences and greater discrimination or stigmatization [30, 35–39]. These consequences are likely individual, but can also be felt at a group level (e.g., prenatal screening of Down syndrome), and the way that they are perceived is dependent on various factors, both internal and external [36].

### Perception of quality of life

There is a general misconception that those living with a genetic condition *necessarily* experience a reduced quality of life. However, research suggests otherwise [10, 34, 40–42]. Many people living with a genetic condition, including “serious” conditions, report comparable well-being as do those without. As demonstrated by Iezzoni et al., most healthcare providers surveyed perceived people living with significant disability as having a worse quality of life than those without disability [43]. This flags an important discrepancy between patient and healthcare provider perspectives, which can negatively impact all facets of care received by those living with a genetic condition (e.g., quality, communication, efficiency, timeliness, equity, safety) [43, 44]. Similarly, Madeo et al. demonstrated that the majority of people living with “serious” genetic conditions, as categorized by professional standards, reported good quality of life and well-being [40]. Amundson argues that one of the key contextual influences that contributes to such erroneous judgments is the focusing illusion (focalism)—whereby a person’s response to a stimulus is “influenced by the context in which the stimulus is perceived” (p. 381) (i.e., focusing on a single aspect of a person’s life rather than all the aspects that comprise one’s life) [34]. Additional considerations that go beyond the scope of this article include distinctions in reported quality of life between those living with physical versus psychological conditions (e.g., anxiety or depression) [45].

To address the lack of conceptual or operational clarity, and to help unpack the notion, this article proposes a set of four core dimensions and four procedural elements that can assist reproductive decision-making for end users (patients, families, and policymakers) regarding seriousness. We claim that these core dimensions and procedural elements can act as a conceptual tool to encourage a common understanding in how we think about seriousness. Ultimately, these considerations would help improve the quality and consistency of decision-making where the seriousness of a genetic condition is a pivotal component in developing and implementing reproductive genomic technologies, in both clinical care and population health settings.

## Considerations around seriousness

Seriousness is a liminal concept that can be used in different areas, by different actors, and for different purposes. For example, for prospective parents, it can be used as shorthand to convey a message about the genetic condition being discussed, but it may not mean the same to everyone. Therefore, a common understanding of what a “serious” genetic condition can entail must be considered in relation to time and place (e.g., country, historical context, culturally sensitive values).

We acknowledge the complexity of this space and that it cannot be a one-size-fits-all approach to defining seriousness. Recognizing the contextual nature of “serious” and how societal understandings may shift over time, it is important that the application of the core dimensions and procedural elements retain flexibility of interpretation around the conception of “serious” to ensure fluidity of its meaning in different contexts over time. Similar to Kukla’s conception of “disease”, “serious” will “take on different meanings and give different answers depending on the goals and stakeholders involved” (p. 136) [28].

As “serious” is actively used in both clinical care and population health, having a set of core dimensions and procedural elements to map the contours of this notion may help to ensure a more coherent and consistent process for understanding the term across contexts. Having a common understanding of its features will serve to facilitate informed decision-making, to promote epistemic justice, to enable comparisons across contexts, and to provide further clarity and guidance for stakeholders. As mentioned at the outset, we propose a set of eight considerations—four core dimensions and four procedural elements—for the appraisal of the seriousness of a genetic condition when it comes to policy and practice regarding reproductive decision-making.

**Fig. 1:**
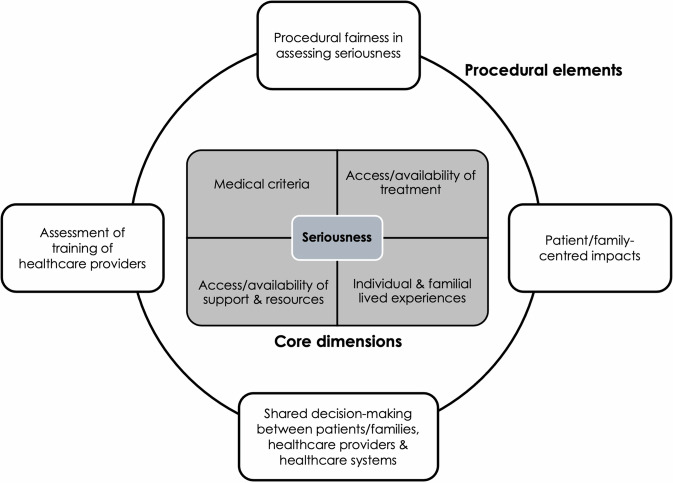
Core dimensions and procedural elements of seriousness. The core dimensions represent considerations related to the theoretical concept of seriousness (i.e., essential factors to consider) and the procedural elements represent how the concept could be implemented in different contexts (i.e., the elements that facilitate discussion of its features or dimensions). These considerations should be understood as complementary and interrelated and should be considered in a proportionate manner, acknowledging different contexts (applications and environments), technologies, cultural and societal values, and levels of risk associated with the technology.

These considerations around seriousness are based on the literature, the authorship team’s previous work [10–13, 23], and the consensus stemming from our international 2023 Brocher Foundation workshop^1^. An initial rapid review and collation of the literature was used to chart existing and emerging literature on seriousness to inform the Brocher workshop application, whilst also identifying potential participants to attend the workshop. Alongside this, publications drawn from the authorship team’s existing knowledge of the field and citation searching was undertaken. The rapid review facilitated the identification of key themes which ultimately became the streams of the workshop: (1) health and healthcare; (2) genomic futures and technologies; (3) lived experience and society; and (4) policy. Following the workshop, the insights and data were collated, and emerging themes were then discussed and refined to identify areas where small clusters of themes could be transformed into a parent theme. The relationships between the themes were then defined and mapped to draft domains. It became clear that there were differences emerging between the core dimensions of the concept of seriousness (i.e., essential factors to consider) and the procedural elements regarding how the concept could be implemented in different contexts (i.e., context in which the dimensions are discussed). The decision was therefore made to separate these considerations in order to distinguish between the theoretical concept of seriousness and the elements that facilitate discussion of its features or dimensions (Fig. 1).

These considerations should be understood as complementary and interrelated (i.e., not mutually exclusive). They should be considered in a proportionate manner, acknowledging different contexts (applications and environments), technologies, cultural and societal values, and levels of risk associated with the technology. The fundamental tensions between clinical care and population health underly decision-making around seriousness and must be accounted for in discussions with a balance between individual values and experience, and clinical manifestations. When conflicts between dimensions arise, subjective/contextual elements are more heavily weighted in the clinical context, whereas objective elements are prioritized in the population context, given the distinct characteristics of each.

There will always be a degree of variability or vagueness around the interpretation of seriousness given the subjective elements associated with how a person perceives and experiences their genetic condition, the variability of symptoms, and the contextual elements that factor in Dive et al. [15]. What is sought by these core dimensions and procedural elements, is to serve as a conceptual tool in establishing consensus in the appraisal (i.e., how we think about the concept), rather than a definition of the concept itself. Despite the difference in how seriousness is being construed in clinical care and population health, the assessment of seriousness remains the same. Therefore, it is the characteristics of the context and its ability to respond to and address different aspects (individual versus population) that can lead to different outcomes of decisions.

Implementation prompts to assist in the application of the core dimensions of seriousness can be found in Table 1. The intention is for these prompts to assist in reflections around and the implementation of the core dimensions below to different contexts. Not all prompts need to be considered, as these will vary depending on the context and purpose of the discussion (e.g., clinical care versus population health). This approach favours ethical and institutional pluralism, in that “no one value should be given preference over all other values in all situations. There are no absolutes. A variety of values must be taken into account in policy decision-making. When values are in conflict they must be balanced off against each other” (p. 21) [46].

**Table 1:** Prompts for the application of the core dimensions of seriousness.

Medical criteria	Access/availability of treatment	Access/availability of support and resources	Individual and familial lived experiences
*Diagnosis* • High morbidity/mortality • Heritability • Type of disability (cognitive, physical, sensory) • Impact on life expectancy (risk of premature death/early mortality)—clinically predictable • Level of penetrance • Certainty of diagnosis & prognosis (single vs. multiple diagnoses) • Disease trajectory (linear vs. cyclical) • Predictability of disease course (degree of risk/uncertainty) • Family history *Clinical manifestations* • Pain & suffering (physical & mental) • Degree of physical & cognitive impairment ◦ Speed of degeneration (if progressive) ◦ Number of body systems affected • Day-to-day functioning (affected autonomy) • Variability of symptoms, expressivity & complexity of phenotype *Timing of onset* • Age of onset *Complications* • Chronic complications • Likelihood of associated/secondary conditions or complications	*Treatment/symptom management* • Availability & access *Goal of treatment* • Curative • Prophylactic • Supportive/symptom management • Experimental *Financial* • Ability to meet costs (out-of-pocket cost of treatment) *Burden of treatment* • Physical • Financial • Interpersonal (impact on family, relationships)	*Resource availability* • Social capital (networks/support) • Financial stability *Resource allocation within the healthcare system* • Mechanisms • Counselling • Benefits/social support • Psycho-social support • Familial support *Financial* • Socio-economic status • Cost of living with condition • Impact on productivity of household (ability to go to work)	*Personal/identity* • Values, preferences & personal identification • Perceived quality of life • Community/identity • Ability to thrive/flourish • Experience with the genetic condition (couple, family, community) *Psycho-social* • Psycho-social burden • Education • Access to paid employment • Impact on health of household (health of parents & siblings) • Impact on the household • Ability to plan for the future • Impact on daily functioning & independent living • Availability & burden of treatment *Socio-cultural* • Ethnicity/culture/language • Religion/spirituality (incl. indigenous) • Societal perceptions/social acceptability • Discrimination/stigmatization (gender/sex, LGBTIQA+, disability, racialized minorities) • Interpretations of health, disease, disability • Historical, legal & political contexts (e.g., conflict affected or legacy of eugenics) *Relationships* • Ability to have & maintain meaningful social relationships (familial, friendships) • Sibling views/perceptions *Built & physical environment* • Accessibility of physical/built environment • Geographic location & proximity to care • Living circumstances (housing quality & location) • Climate

For ease of reference, prompts have been placed into the best-aligned category with the understanding that what is in one category may also fall into another (i.e., not mutually exclusive) and these core dimensions are understood as complementary and interrelated.

### Core dimensions

#### Medical criteria

The objective medical aspects or clinical manifestations (i.e., related to diagnosis, complications, side effects of treatment, timing of onset) of a genetic condition must be considered in our understanding/application of seriousness in both clinical care and population health with greater weight attributed to the latter. These include factors such as: morbidity, impact on life expectancy (risk of premature death/early mortality), level of penetrance, variability and intensity of symptoms, degree of pain/suffering, and age of onset. Whilst identification of genetic conditions is becoming increasingly accessible in prenatal testing contexts (whole genome/exome sequencing), factors such as incomplete penetrance and variable expressivity of seemingly pathogenic variants, together with a lack of phenotype can render seriousness extremely difficult to decipher [47]. Importantly, the limitations of the medical model in being able to accurately and completely bridge the gap between clinical diagnoses or pathogenic variants, and the way they “show up in the world” in human lives, bodies, and experiences (lived reality) must be made clear.

#### Access/availability of treatment

There are implications from an equity perspective for distributive justice and the way we think about using new reproductive genomic technologies or how the healthcare system prioritizes certain treatments that raises questions of resource allocation and access to treatments that need to be considered by governments.

The availability of a treatment (curative or supportive) to mediate the symptoms of a given genetic condition and the extent to which these are accessible to those living with the condition must factor into considerations around seriousness, including the burden of undergoing the treatment (physical, financial, interpersonal, etc.) and the availability of resources.

It is a government’s responsibility to prioritize available resources for the health of its population. To do so, objective decisions based on measurable qualifiers are the norm. When determining access parameters for the use of reproductive genomic technologies at the population level, these parameters should be considered for specific policy purposes (e.g., to determine access, funding, or justification for the use) and executed to maximize utility. Such decisions are not meant to predict ex post experiences of a genetic condition’s seriousness in individuals.

#### Access/availability of support and resources

It is important to reflect on what types of mechanisms or support (e.g., counselling, social support, resources, familial support, infrastructure) can be built-in or integrated into the healthcare system for people living with different genetic conditions, as these will determine how “serious” some conditions will be. For example, the experience of living with a spinal cord injury, and the appraisal of its seriousness, in a country where roads, resources, and assistive technologies are available will differ significantly from that of someone living with the same condition in a country where such supportive measures are unavailable [22].

Policymaking is also “influenced by local contextual considerations, the views of the wider scientific community and even policy decisions made in other jurisdictions, both nationally and internationally” (p. 95) [27]. When discussing population health and decision-making, this encompasses a variety of actors and entities having various levels or degrees of obligations, authority, and autonomy, which can make it a challenge to navigate this space. It also further supports the need for a common understanding of the relevant factors and dimensions in order to facilitate a coherent and consistent consideration of seriousness. However, we recognize that reaching consensus on this concept may be difficult, and that decisions may vary across jurisdictions.

There is a general push towards certain genetic conditions being categorized as “serious” in an attempt to make them a social priority (i.e., allow, fund, or justify different outcomes) and allow access to additional support. This contrasts with the lived experiences of some people with genetic conditions, where the push may be rather to see conditions as less “serious” so as to expand the social acceptability of diverse ways of living [48].

#### Individual and familial lived experiences

A purely medical or objective view of seriousness is too narrow to capture the meaningfulness of a given genetic condition (i.e., how it permeates through a person’s life, and the meaning assigned to that permeation). The lived experiences of people and families with genetic conditions is a rich resource that ought to be considered at all levels of decision-making to different degrees. It is dynamic and can evolve over time with a person’s subjective experience of the day-to-day.

Lived experience is notably difficult to capture, characterize, measure, and transfer between persons as it is inherently unstable and constantly being revised [49], similar to medical knowledge. However, this is not a justification for its exclusion in considerations of what makes a condition “serious”. Indeed, integrating diverse accounts of lived experience (individual and familial) into seriousness discussions allows for a range of perspectives and examples of the subjective factors that comprise the experience of living with a genetic condition both for clinical care and population health.

All lived experiences must be contextualized within the broader factors of systemic inequalities, socio-economic conditions, and historical contexts. Various subjective factors at play offer a richness to lived experience—values, preferences, social context, cultural context, religious context, economic context, mental/emotional dimensions, political context, physical/built environment—which can mediate the perception of seriousness. This highlights the importance of considering values as these play a role in how people attach moral value to a condition and will have a direct impact on their perception of seriousness. The impact of socio-economic status and the amount of social support a person has access to can also have a direct impact on how they perceive their condition [50]. Capturing a person’s experience living with a genetic condition is essential to capturing the full picture of a condition and should be considered in parallel with medical criteria.

Disability can be identity-constituting (e.g., earlier onset, stable conditions versus late onset, painful, degenerative life-threatening or life-limiting conditions). This can be either positively or negatively construed depending on the person. This includes family expectations or structure, extent towards which the condition fits with the accepted norm, and whether the condition is associated with community, culture, and identity (e.g., deafness or achondroplasia). Therefore, it is important to consider how society and culture frame a condition, as this can have an impact on a person’s mental well-being and how they ultimately perceive their experience living with the condition (e.g., stigma plays a big role in different societies) [51, 52].

Familial lived experience equally contributes to considerations around the seriousness of a genetic condition and the potential impact this could have on family life. Factors at play within the familial context include familial values and circumstances; family resilience (i.e., ability to cope with or raise a child with a genetic condition); the impact on other siblings and familial/kinship relationships; the burden and pressure of caring for a child with special needs; financial stability; competing responsibilities; social exclusion; and familial guilt or stigma [52–55]. Moreover, the personal experience of having a child or knowing someone with a genetic condition may influence how the family considers what it means to have such a condition, as well as their perception of a child’s expected quality of life and the impact this may have on the family’s overall quality of life [52–54].

The nature of a person’s lived experience, with contrasting perspectives between those living with generally static congenital conditions and those who either acquire their condition unexpectedly or whose condition worsens over time will also influence attitudes towards seriousness [56]. Boardman and Clark reported that when assessing their own well-being, “physical and psychological adaptation to the condition, including its incorporation within personal identity” (p. 168) played an important role for people living with genetic conditions [10]. Those who have integrated their condition into their sense of self, facilitated and accelerated by its early onset, were less likely to see their condition as “serious” [10, 29, 34, 57, 58]. As such, the timing of a condition’s onset impacts perception of quality of life, with later onset conditions being perceived by those living with them as resulting in a lower quality of life [10]. Whether a person has integrated their condition/diagnosis with their sense of self (i.e., identity) directly impacts their perception of how “serious” their condition is [59]. Therefore, the way seriousness is calibrated for a given person relies heavily on their lived experiences.

Moreover, there is discrepancy between objectively determined quality of life and the subjective quality of life experienced by those living with a genetic condition. This highlights the importance of patient testimony and ensuring people are given the time and space to articulate and communicate their own individual experiential knowledge of “serious”. Those living with genetic conditions are experts of their own condition, and having access to the diversity of experiential knowing and the subjective elements that comprise the lived experience of a genetic condition are essential to incorporate in the appraisal of seriousness—both at a clinical and population level [30, 60–63].

Healthcare providers, parents, and people with lived experience of a genetic condition may be measuring very different things when discussing a condition and the impact on one’s life [10, 43]. Therefore, it is important to identify where there are differences between how people define and experience seriousness and how they appear within medical practice. However, it is also important to determine whether there are features that people identify as having a “serious” impact on their lives, ones that are not foreseen or accounted for from a clinical perspective (e.g., the differences in acceptance between early and late onset genetic conditions or social stigma associated with a condition).

### Procedural dimensions

#### Procedural fairness in assessing seriousness

Decision-making around whether to fund or offer access to reproductive genomic technologies requires transparency around the motivations and objectives behind the decision, as well as setting clear thresholds on permitted uses. Care ought to be taken in considering different socio-political contexts specific to a country or society when making decisions that have impacts at the population level. Moreover, clear language is essential when drafting laws and policies so as to facilitate coherent and consistent decisions; yet, responsive and flexible considering different contexts and age groups. This is particularly relevant in population health as the purpose for which reproductive genomic technologies are being employed is directly tied to thinking about seriousness in relation to these purposes.

Goals and objectives should be evidence-based and include data from quantitative, longitudinal, qualitative (sociological/psychological), as well as deliberative research that may help to capture the diversity of people’s experiences and highlight the complexity of decisions made in a given context. Evidence-based information can also help to mitigate existing systemic biases and bring awareness to other biases held by both healthcare providers and policymakers.

Tension can exist between a policy categorizing a genetic condition as “serious” from a population perspective, and how a person living with the condition internalizes their experience and incorporates it into their identity (i.e., reject the categorization) [57]. It is important to recognize that by categorizing a condition as “serious”, we risk perpetuating certain societal views (e.g., eugenics, ableism) or leading to greater stigmatization by the public, and this may be problematic and even dangerous. Although not entirely avoidable, policymakers ought to be aware of this risk and consider potential repercussions as they develop policy and make decisions that impact the population.

#### Patient/family-centred impacts

The seriousness of a genetic condition in a particular person is influenced by many different factors in their lives, such as experiential factors, values, preferences, personal identification with the condition, and socio-cultural context. This richness cannot be collapsed into a generalization about the condition by aggregating those experiences. Attention must be given to these factors and a person must be considered within their context or environment, beyond simply medical criteria and clinical measures [57, 64].

There is also a need to recognize that the impact of a genetic condition may not be limited to an individual, but their family and community as well. Considerations around individual (lived) experiences of what seems manageable or acceptable for the person themselves is essential but must also include considerations around their close environment and capacity to deal with important changes in life, their individual socio-economic situation, cultural and societal background, and required/available support (e.g., a financially secure couple in their 40s considering prenatal diagnosis in their first pregnancy is very different from that same diagnosis in a third pregnancy of a younger person living in poverty).

#### Shared decision-making between patients/families, healthcare providers and healthcare systems

Clinical services that involve discussion about reproductive genomic technologies need to involve the capacity for listening and shared meaning making about the impact of the decision for the prospective parents, the future child, and their family. The goal is to foster dialogue and explore seriousness and what a diagnosis may imply for a person’s life from their perspective, given their personal experience and context.

Moreover, the way information is conveyed to the person should be manageable and understandable, and sufficient time should be provided for them to process, reflect, and discuss. There needs to be an openness in how information is presented (i.e., avoid judgments about seriousness and rather point to what may be constitutive of it). It is important to remember that the prospective parents may have a very different notion or understanding of seriousness than the healthcare providers caring for them. The prospective parents’ absorptive capacity must also be considered, as they may be in a distressed state at the time this is being discussed with them. This can lead to difficulties in engagement following an unanticipated diagnosis [65]. In the prenatal context, seriousness may also be extremely difficult to predict and to explain to prospective parents with no experience of the condition. Therefore, the use of these terms should be accompanied by neutral, balanced examples and a non-directive approach to communicate the range and probability of likely impacts, including direct experiences and views of people living with a genetic condition and their families [30, 53].

Particularly in population health contexts, there is also an element of education or socialization around the genetic condition or concept that is required to ensure that citizens and prospective parents feel empowered to participate in the conversation, articulate their values/beliefs, and make decisions [25]. Potential risks around how information is communicated can have societal implications and affect participation. Therefore, it is important to recognize that there may be a risk of perpetuating or shifting certain societal norms/views that could be problematic or perpetuate epistemic injustice (e.g., ableism, eugenics, stigmatization) and consider mechanisms to help mitigate such risks at the population level.

#### Assessment of training of healthcare providers

It is important for governments and healthcare systems to ensure that healthcare and population health providers have access to appropriate and adequate training in how to engage with patients and effectively counsel them (balanced or neutral approach that includes the narrative accounts of those living with conditions). A growing concern is the limited availability of adequate counselling [66–69]. With a shift towards whole genome sequencing and expanded screening panels, a question of scalability arises given the lack of genetic counsellors and the resources that would be required.

Moreover, it is essential for healthcare providers to be able to recognize and mitigate potential biases or public value judgments when providing support for people living with different kinds of genetic conditions or impairments, and consider how these may impact the delivery of care [40].

## Conclusion

Use of the concept of seriousness in reproductive decision-making is one of the most ethically complex uses of the term, as it influences not only prospective parents’ reproductive decisions and family planning, but also has a direct influence on societal decisions around who is born. Although traditionally distinct, the rise of predictive and precision medicine is blurring the boundaries of policy and practice in the clinical and population contexts as genomics is moving out of the clinic and into the public health sphere. This is even more relevant in the context of reproductive genomic technologies given their expansion into screening, rendering them more accessible to a larger portion of the population.

The core dimensions of seriousness and the procedural elements that are required for their consideration, as outlined in this article, are intended to serve as a conceptual tool to support discussions around the meaning of seriousness. Drawing on previous work, the wider literature, and a consensus building workshop, the core dimensions and procedural elements were designed in such a way as to allow them to be adapted in different contexts. Where local laws, policies, and regulations exist around access to and use of reproductive genomic technologies, for example, these would need to be taken into account and frame considerations of what seriousness can mean in that particular social, legal, and environmental context. The weighting of each dimension is also context dependent, with certain dimensions having a greater relevance to clinical care and others to population health. This allows for an operational and adaptive approach to understandings of seriousness in different contexts. Despite varied weighting, both core dimensions and procedural elements must be considered in the appraisal of seriousness, and this, in both the clinical and population setting. In clinical care, healthcare providers can use the core dimensions and procedural elements to help them think about how to frame discussions around seriousness with prospective parents and make judgments about how much information they can cope with at different points in time [32]. In the context of population health, the use of seriousness as a criterion or threshold in determining the permissibility of or access to emerging reproductive genomic technologies epitomizes what a country considers is necessary to ensure the right to the highest attainable standard of health for both its actual and future citizens [1, 11, 70]. Thus, the core dimensions and procedural elements serve as a conceptual tool to support consensus in the components necessary for its appraisal, rather than a definition of seriousness.

This article offers a starting point for discussions on seriousness. Next steps include exploring how the core dimensions and procedural elements interact and are applied, as well as their relevance beyond genomic and reproductive medicine. Such an exploration will necessarily include providing comparative examples that highlight the benefits and challenges of the proposed approach, as well as considering how they could help inform training for healthcare providers and policymakers. We encourage further discussions and deliberations around these core dimensions and procedural elements, such as how they could be expanded or collapsed, and what potential uses may look like in other contexts, including beyond the dominant western paradigm.

Indeed, as genomics increasingly enters both population health programs such as newborn and cancer screening and is integrated into clinical practice, the list of conditions that can be identified is growing and reliance on a panel of monogenic serious conditions may no longer be adequate. The core dimensions and procedural elements of seriousness are useful for framing discussions in both clinical care and population health. Whilst developed with reproductive genomic technologies in mind, they may have relevance beyond genomics (e.g., to conditions identified antenatally without genetic testing, such as spina bifida) and beyond reproduction (e.g., resource allocation and screening programs for adults). Further work is needed to explore these other areas and applications.

## Footnotes

1 A 2.5-day workshop entitled “Conceptualising Severity in a Genomic Age” was hosted and supported by the Brocher Foundation in Hermance, Switzerland from June 13 to 15, 2023. It included 22 international participants from Australia, Belgium, Canada, France, Germany, the Netherlands, Norway, Switzerland, the United Kingdom, and the United States. Participants represented a wide range of career stages and disciplinary backgrounds (e.g., academics, patients/patient advocacy groups, clinicians/professionals, and policy advisors/regulatory groups) and were invited based on their experience working in areas where the notion of “serious” condition is critical.

## References

[1] Barra M, Broqvist M, Gustavsson E, Henriksson M, Juth N, Sandman L, et al. Severity as a priority setting criterion: setting a challenging research agenda. Health Care Anal. 2020;28:25–44. 31119609 10.1007/s10728-019-00371-z PMC7045747

[2] Nord E, Johansen R. Concerns for severity in priority setting in health care: a review of trade-off data in preference studies and implications for societal willingness to pay for a QALY. Health Policy. 2014;116:281–8. 24690334 10.1016/j.healthpol.2014.02.009

[3] European Commission, Directorate-General for Research and Innovation. European group on ethics in science and new technologies opinion on the ethics of genome editing. Luxembourg: Publications Office of the European Union; 2021. p. 110. https://data.europa.eu/doi/10.2777/659034

[4] Heritable Human Genome Editing. Washington, D.C.: National Academies Press; 2020. p. 238. https://www.nap.edu/catalog/25665 32897669

[5] Comité consultatif national d’éthique pour les sciences de la vie et de la santé. Avis 133 – Enjeux éthiques des modifications ciblées du génome: entre espoir et vigilance. 2019. p. 46. https://www.ccne-ethique.fr/sites/default/files/2021-02/avis_133_-_ad_final.pdf

[6] German Ethics Council. Intervening in the human germline: opinion (executive summary & recommendations). Berlin: German Ethics Council; 2019. p. 60.

[7] Commission de l’éthique en science et en technologie. Bébés génétiquement modifiés: Enjeux éthiques soulevés par la modification génétique des cellules germinales et des embryons. Québec: Gouvernement du Québec; 2019. p. 110. https://www.ethique.gouv.qc.ca/fr/publications/modification-genetique-de-la-lignee-germinale/

[8] Nuffield Council on Bioethics. Genome editing and human reproduction: social and ethical issues. London, UK: Nuffield Council on Bioethics; 2018. p. 183. https://www.nuffieldbioethics.org/publications/genome-editing-and-human-reproduction

[9] Human genome editing: science, ethics, and governance. Washington, D.C.: National Academies Press; 2017. https://www.nap.edu/catalog/24623 28796468

[10] Boardman FK, Clark CC. What is a ‘serious’ genetic condition? The perceptions of people living with genetic conditions. Eur J Hum Genet. 2022;30:160–9. 34565797 10.1038/s41431-021-00962-2 PMC8821585

[11] Kleiderman E, Ravitsky V, Knoppers BM. The ‘serious’ factor in germline modification. J Med Ethics. 2019 ;45:508–13. 31326898 10.1136/medethics-2019-105436 PMC6820154

[12] Newson AJ, Dive L. Taking seriousness seriously in genomic health. Eur J Hum Genet. 2022;30:140–1. 34782753 10.1038/s41431-021-01002-9 PMC8821609

[13] Wertz DC, Knoppers BM. Serious genetic disorders: can or should they be defined? Am J Med Genet. 2002;108:29–35. 11857546 10.1002/ajmg.10212

[14] Savell K, Karpin I. The meaning of “serious disability” in the legal regulation of prenatal and neonatal decision-making. J Law Med. 2008;16:233–45. Oct 19010002

[15] Dive L, Archibald AD, Freeman L, Newson AJ. How should severity be understood in the context of reproductive genetic carrier screening? Bioethics. 2023;37:359–66. 36744627 10.1111/bioe.13136

[16] Definition of SERIOUS. In: Merriam-Webster. 2024. https://www.merriam-webster.com/dictionary/serious

[17] Definition of SEVERE. In: Merriam-Webster. 2024. https://www.merriam-webster.com/dictionary/severe

[18] Arjunan A, Bellerose H, Torres R, Ben‐Shachar R, Hoffman JD, Angle B, et al. Evaluation and classification of severity for 176 genes on an expanded carrier screening panel. Prenat Diagn. 2020;40:1246–57. 32474937 10.1002/pd.5762 PMC7540025

[19] Korngiebel DM, McMullen CK, Amendola LM, Berg JS, Davis JV, Gilmore MJ, et al. Generating a taxonomy for genetic conditions relevant to reproductive planning. Am J Med Genet Part A. 2016;170:565–73. 26889673 10.1002/ajmg.a.37513 PMC4860293

[20] Lazarin GA, Hawthorne F, Collins NS, Platt EA, Evans EA, Haque IS. Systematic classification of disease severity for evaluation of expanded carrier screening panels. PLoS ONE. 2014;9:e114391. 25494330 10.1371/journal.pone.0114391 PMC4262393

[21] Dive L, Archibald AD, Newson AJ. Ethical considerations in gene selection for reproductive carrier screening. Hum Genet. 2022;141:1003–12. 34426854 10.1007/s00439-021-02341-9 PMC9160090

[22] Allotey P, Reidpath D, Kouamé A, Cummins R. The DALY, context and the determinants of the severity of disease: an exploratory comparison of paraplegia in Australia and Cameroon. Soc Sci Med. 2003;57:949–58. 12850119 10.1016/s0277-9536(02)00463-x

[23] Kleiderman E, Roy MC, Knoppers BM, Ravitsky V. BioNews. 2021. A “serious” threshold for genomic technologies – context counts! https://www.progress.org.uk/a-serious-threshold-for-genomic-technologies-context-counts/

[24] Dive L, Newson AJ. Ethics of reproductive genetic carrier screening: from the clinic to the population. Public Health Ethics. 2021;14:202–17. 34650621 10.1093/phe/phab017 PMC8510688

[25] Armstrong KA, Metlay JP. Annals clinical decision making: communicating risk and engaging patients in shared decision making. Ann Intern Med. 2020;172:688–92. 32311739 10.7326/M19-3495

[26] Di Mattei V, Ferrari F, Perego G, Tobia V, Mauro F, Candiani M. Decision-making factors in prenatal testing: a systematic review. Health Psychol Open. 2021;8:2055102920987455. 33489303 10.1177/2055102920987455 PMC7809316

[27] Andermann A, Blancquaert I, Déry V. Genetic screening: a conceptual framework for programmes and policy-making. J Health Serv Res Policy. 2010;15:90–7. 20176664 10.1258/jhsrp.2009.009084

[28] Kukla QR. What counts as a disease, and why does it matter? J Philos Disabil. 2022. https://www.pdcnet.org/pdc/bvdb.nsf/purchase?openform&fp=jpd&id=jpd_2022_0999_6_6_13

[29] Freeman L, Righetti S, Delatycki MB, Scully JL, Kirk EP. The views of people with a lived experience of deafness and the general public regarding genetic testing for deafness in the reproductive setting: a systematic review. Genet Med. 2022;24:1803–13. 35659827 10.1016/j.gim.2022.05.005

[30] Gollust SE, Thompson RE, Gooding HC, Biesecker BB. Living with achondroplasia: attitudes toward population screening and correlation with quality of life. Prenat Diagn. 2003;23:1003–8. 14663838 10.1002/pd.743

[31] Shakespeare T. Choices and rights: eugenics, genetics and disability equality. Disabil Soc. 1998;13:665–81. 11660717 10.1080/09687599826452

[32] Boardman F, Thomas G. Expressivist objections to prenatal screening and testing: perceptions of people living with disability. Sociol Health Illn. 2023;45:1223–41. 36181509 10.1111/1467-9566.13559

[33] Dive L, Newson AJ. Reproductive carrier screening: responding to the eugenics critique. J Med Ethics. 2022 ;48:1060–7. 34244346 10.1136/medethics-2021-107343 PMC9726954

[34] Amundson R. Quality of life, disability, and hedonic psychology. J Theory Soc Behav. 2010;40:374–92.

[35] Werkhoven S, Anderson JH, Robeyns IAM. Who benefits from diagnostic labels for developmental disorders? Dev Med Child Neurol. 2022;64:944–9. 35191027 10.1111/dmcn.15177 PMC9306602

[36] Sims R, Michaleff ZA, Glasziou P, Thomas R. Consequences of a diagnostic label: a systematic scoping review and thematic framework. Front Public Health. 2021;9:1–26. 10.3389/fpubh.2021.725877 PMC8727520 35004561

[37] Wright A, Jorm AF, Mackinnon AJ. Labeling of mental disorders and stigma in young people. Soc Sci Med. 2011;73:498–506. 21794967 10.1016/j.socscimed.2011.06.015

[38] Parens E, Asch A. Disability rights critique of prenatal genetic testing: reflections and recommendations. Ment Retard Dev Disabil Res Rev. 2003;9:40–7. 12587137 10.1002/mrdd.10056

[39] Shakespeare T. Nasty, brutish, and short? on the predicament of disability and embodiment. In: Schmitz B, Felder F, Bickenbach JE, editors. Disability and the good human life. Cambridge: Cambridge University Press; 2013. pp. 93–112. (Cambridge Disability Law and Policy Series). https://www.cambridge.org/core/books/disability-and-the-good-human-life/nasty-brutish-and-short-on-the-predicament-of-disability-and-embodiment/61BFDFDF0F43C6DBFAA4CE5EF01DCBD7

[40] Madeo AC, Biesecker BB, Brasington C, Erby LH, Peters KF. The relationship between the genetic counseling profession and the disability community: a commentary. Am J Med Genet Part A. 2011;155:1777–85. 10.1002/ajmg.a.34054 PMC5240749 21567935

[41] Viemerö V, Krause C. Quality of life in individuals with physical disabilities. Psychother Psychosom. 1998;67:317–22. 9817953 10.1159/000012297

[42] Albrecht GL, Devlieger PJ. The disability paradox: high quality of life against all odds. Soc Sci Med. 1999;48:977–88. 10390038 10.1016/s0277-9536(98)00411-0

[43] Iezzoni LI, Rao SR, Ressalam J, Bolcic-Jankovic D, Agaronnik ND, Donelan K, et al. Physicians’ perceptions of people with disability and their health care. Health Aff. 2021;40:297–306. 10.1377/hlthaff.2020.01452 PMC8722582 33523739

[44] Hannawa AF, Wu AW, Kolyada A, Potemkina A, Donaldson LJ. The aspects of healthcare quality that are important to health professionals and patients: a qualitative study. Patient Educ Couns. 2022;105:1561–70. 34711447 10.1016/j.pec.2021.10.016

[45] Berghöfer A, Martin L, Hense S, Weinmann S, Roll S. Quality of life in patients with severe mental illness: a cross-sectional survey in an integrated outpatient health care model. Qual Life Res. 2020;29:2073–87. 32170584 10.1007/s11136-020-02470-0 PMC7363717

[46] Finsterbusch K. How should policy decisions be made? Impact Assess. 1989;7:17–24.

[47] Nov-Klaiman T, Bowman-Smart H, Horn R. Negotiating severity behind the scenes: prenatal testing in Germany. Eur J Hum Genet. 2024:1–6. 10.1038/s41431-024-01612-z PMC11839981 38678162

[48] Brady G, Franklin A, Collective R. ‘I am more than just my label’: rights, fights, validation and negotiation. Exploring theoretical debates on childhood disability with disabled young people. Sociol Health Illn. 2023;45:1376–92. 37341685 10.1111/1467-9566.13678

[49] Boardman FK. Experience as knowledge: disability, distillation and (reprogenetic) decision-making. Soc Sci Med. 2017;191:186–93. 28926777 10.1016/j.socscimed.2017.09.013 PMC7610975

[50] Freeman L, Delatycki MB, Leach Scully J, Kirk EP. Views of reproductive genetic carrier screening participants regarding screening for genes associated with non‐syndromic hearing loss. Prenat Diagn. 2022;42:1658–66. 36289583 10.1002/pd.6253 PMC10100309

[51] Boardman FK, Hale R. “I didn’t take it too seriously because I’d just never heard of it”: experiential knowledge and genetic screening for thalassaemia in the UK. J Genet Couns. 2019;28:141–54. 30629758 10.1002/jgc4.1042 PMC7814888

[52] Boardman FK. Attitudes toward population screening among people living with fragile X syndrome in the UK: ‘I wouldn’t wish him away, I’d just wish his fragile X syndrome away’. J Genet Couns. 2021;30:85–97. 33184995 10.1002/jgc4.1355 PMC7894324

[53] Nov-Klaiman T, Frisman M, Raz AE, Rehmann-Sutter C. Views on disability and prenatal testing among families with Down syndrome and disability activists: a comparative analysis of interviews from Germany and Israel. Soc Sci Med. 2022;303:115021. 35588654 10.1016/j.socscimed.2022.115021

[54] Blakeley C, Smith DM, Johnstone ED, Wittkowski A. Parental decision-making following a prenatal diagnosis that is lethal, life-limiting, or has long term implications for the future child and family: a meta-synthesis of qualitative literature. BMC Med Ethics. 2019;20:56. 31395047 10.1186/s12910-019-0393-7 PMC6688313

[55] Dive L, Laberge AM, Freeman L, Bunnik EM. Beyond severity: utility as a criterion for setting the scope of RGCS. Eur J Hum Genet. 2024:1–5. 10.1038/s41431-024-01640-9 PMC11840050 38811715

[56] Boardman FK, Young PJ, Warren O, Griffiths FE. The role of experiential knowledge within attitudes towards genetic carrier screening: a comparison of people with and without experience of spinal muscular atrophy. Health Expect. 2018;21:201–11. 28703871 10.1111/hex.12602 PMC5750730

[57] Boardman FK, Young PJ, Griffiths FE. Impairment experiences, identity and attitudes towards genetic screening: the views of people with spinal muscular atrophy. J Genet Couns. 2018;27:69–84. 28664217 10.1007/s10897-017-0122-7 PMC5794814

[58] Watson N. Well, I know this is going to sound very strange to you, but I don’t see myself as a disabled person: identity and disability. Disabil Soc. 2002;17:509–27.

[59] Paul DB. Imagining life with a genetic disorder: the challenge of evaluating health states that exist from birth. OBM Genet. 2021;5:1–18.

[60] Carel H, Kidd IJ. Epistemic injustice in medicine and healthcare. In: The Routledge handbook of epistemic injustice. Abingdon: Routledge Handbooks Online; 2017. pp. 336–47. https://www.routledgehandbooks.com/doi/10.4324/9781315212043.ch32

[61] Scrutton AP. Epistemic injustice and mental illness. In: The Routledge handbook of epistemic injustice. Abingdon: Routledge Handbooks Online; 2017. pp. 347–56. https://www.routledgehandbooks.com/doi/10.4324/9781315212043.ch33

[62] Petersen A. The best experts: the narratives of those who have a genetic condition. Soc Sci Med. 2006;63:32–42. Jul 1 16431006 10.1016/j.socscimed.2005.11.068

[63] Boardman FK. Knowledge is power? The role of experiential knowledge in genetically ‘risky’ reproductive decisions. Sociol Health Illn. 2014;36:137–50. 24111508 10.1111/1467-9566.12048

[64] Brady NC, Bruce S, Goldman A, Erickson K, Mineo B, Ogletree BT, et al. Communication services and supports for individuals with severe disabilities: guidance for assessment and intervention. Am J Intellect Dev Disabil. 2016;121:121–38. 26914467 10.1352/1944-7558-121.2.121 PMC4770561

[65] White AL, Boardman F, McNiven A, Locock L, Hinton L. Absorbing it all: a meta-ethnography of parents’ unfolding experiences of newborn screening. Soc Sci Med. 2021;287:114367. 34534781 10.1016/j.socscimed.2021.114367 PMC8505793

[66] Berninger T, Nusbaum R, Redlinger-Grosse K, Davis C, Reiser C. A narrative literature review: growing the workforce through increased fieldwork capacity in genetic counseling training programs. J Genet Couns. 2021;30:574–87. 33124158 10.1002/jgc4.1346

[67] Stoll K, Kubendran S, Cohen SA. The past, present and future of service delivery in genetic counseling: keeping up in the era of precision medicine. Am J Med Genet Part C Semin Med Genet. 2018;178:24–37. 29512888 10.1002/ajmg.c.31602

[68] Hoskovec JM, Bennett RL, Carey ME, DaVanzo JE, Dougherty M, Hahn SE, et al. Projecting the supply and demand for certified genetic counselors: a workforce study. J Genet Couns. 2018;27:16–20. 29052810 10.1007/s10897-017-0158-8

[69] Brunger JW, Matthews AL, Smith RHJ, Robin NH. Genetic testing and genetic counseling for deafness: the future is here. Laryngoscope. 2001;111:715–8. 11359145 10.1097/00005537-200104000-00027

[70] Stenmarck MS, Jølstad B, Baker R, Whitehurst DGT, Barra M. A severely fragmented concept: uncovering citizens’ subjective accounts of severity of illness. Soc Sci Med. 2023;330:1–9. 10.1016/j.socscimed.2023.116046 37392648

